# Global Provisioning of Red Meat for Flexitarian Diets

**DOI:** 10.3389/fnut.2018.00050

**Published:** 2018-06-14

**Authors:** Talia M. Hicks, Scott O. Knowles, Mustafa M. Farouk

**Affiliations:** ^1^Food Assurance and Meat Quality, Food and Bio-based Products Group, AgResearch Limited, Hamilton, New Zealand; ^2^Food Nutrition and Health, Food and Bio-based Products Group, AgResearch Limited, Palmerston North, New Zealand

**Keywords:** flexitarian, red meat, offal, food security, nutrition, production, waste

## Abstract

Although not always labeled as such, flexitarianism is the default lifestyle for much of the world, whereby meals based on plant materials provide the bulk of people's calories. The rich nutrition of meat and animal products is often the lynchpin of these diets, even when only consumed occasionally. It provides forms and concentrations of essential proteins, lipids, and micronutrients that are otherwise scarce. However, the production of this meat is resource intensive. It requires large quantities of arable land and water, and typically has lower conversion efficiency of farm inputs to edible outputs compared with crops, poultry, aquaculture, dairy, and eggs. An additional complication is that the quantity of ancillary products produced during slaughterhouse operations is large and underutilized. Each year, approximately 190 million metric tons (MMT) of red meat, including pork, lamb, sheep, veal, beef, and goats are produced globally, half of which will be consumed by less than 25% of the population living in developed countries. With demand for meat expected to exceed 376 MMT by 2030, an increase in the adoption of plant-based diets presents an opportunity for the world to re-evaluate how meat can be sustainably produced, with greater emphasis on animal welfare, nutritional value, product safety, better utilization, and distribution channels. In this article we consider the role meat plays in the modern diet, its production and consumption, opportunities to improve utilization of the animal, the benefits of incorporating a diverse range of red meat into diets, and the strategies that the meat industry should consider in response to flexitarianism.

## Introduction

### Past and present food production

Hominins began incorporating animal products into their diet at least 2.6 million years ago (1). Consumption of small game, eggs, fish, bone marrow, and carrion may have been pivotal in the evolution of humans (2–4), and potentially drove the success of our species as it dispersed from Africa (5). Humans are omnivorous consumers, as evidenced by comparison to carnivores and herbivores (6, 7). Differences in dental and bowel morphology show how physiology accommodates diets that include nutritionally dense, animal-derived foods, rather than solely leaves, fruits, seeds, and cereals (4, 6, 8–10).

Animal products were probably scarce resources for ancient populations, until ca. 12,000 years ago when the introduction of agriculture, the cultivation of plants and the domestication of livestock revolutionized the way people lived and ate (7, 11). Early approaches to agriculture included pastoralism and mixed crop-livestock strategies, from which all modern farming practices are derived. Pastoral systems relied on mobile methods of livestock management, following the seasonal migration of herd animals when necessary (12). Mixed farming, or “agro-pastoralism,” implemented permanent infrastructure such as buildings and fenced fields for confining the animals. Livestock became integrated into the processes of crop cultivation in order to plow the land and fertilize it, while additionally producing wool, milk, edible tissues and hides (12). These new agricultural-based communities roamed less to find their food, and thus offered stability for civilizations to take root (13).

The expansion of industrialized agriculture allowed the global population to soar, from some four million people 10,000 years ago, to more than seven billion today (14, 15). Methods diverged from traditional farming, and modern intensive production of crops and livestock is now commonplace in developed countries (16, 17). Enough food is being produced to satisfy global caloric demand, at least in principle. In 2013, food supply exceeded 4,876 million metric tons (MMT), equating to 2,880 kcal/person/day, including 302 MMT of meat providing 237 kcal/person/day (18). Yet malnourishment is widespread. Two billion people lack adequate energy, protein or key micronutrients such as iron and vitamin A, and more than 800 million go hungry each day (19). It would appear that the hurdle is not growing sufficient food, but rather getting it to those in need. The problem will likely be exacerbated as populations grow, demand rises, and more countries adopt Westernized eating habits (20–22).

### Meat and food security

Everyone deserves continuous access to sufficient, safe and nutritious food. Securing the supply will require improving crop yields and increasing production limits, minimizing food losses, and recovering unnecessary waste, all while managing the social and environmental impacts of urbanization, competition for land and water, habitat degradation and biodiversity loss (22–24). In this context the industrialized production of meat will be complex and contentious to maintain. Its contribution to diet quality must be worth the cost in resources and otherwise edible feeds (18, 25–27).

In this article we consider how incorporating meat into diets, even in modest amounts, can facilitate and improve food security. We describe how the current meat industry fits into world economies, some of the opportunities to improve carcass utilization, new interest in the diversity of consumed species, and how nutrient-dense foods originating from animals can fill gaps in varied diets. Substantive additional topics, such as calculating the environmental impact of production and the unsettled arguments about the health risks of meat consumption, are not discussed here. These have been examined in detail elsewhere, including those investigating environmental impacts (28–31), reviews of colorectal cancer risk (32–36) and other types of cancer and negative health outcomes such as type 2 diabetes and cardiovascular diseases (37–39). Meat consumption has accompanied human survival for centuries, but it can no longer be taken for granted in modern diets. Its position of importance and privilege is being challenged by the increasing number of people who identify as flexitarians (30, 40).

## Global production and consumption of meat

### What is produced

What is considered to be meat varies across cultures, is characterized inconsistently by nutrition scientists vs. meat scientists, and lacks a standardized lexicon to organize its classification and accounting (41). “Red” meat typically includes muscle and edible offal from cattle, sheep, deer, goats, and sometimes pigs (42). Offal is the organs, tissues or other parts of the animal (excluding fat) that are separated during carcass preparation, and what qualifies as edible varies from country to country. It is a co-product of slaughtered animals and can comprise 10–15% of the liveweight (43, 44). Offal may be aggregated with muscle meat production in national statistics, depending on local definitions of “dressed carcass weight,” which makes international production of co-products difficult to track and calculate (44).

Each year approximately 190 MMT of red meat is produced globally, half of which will be consumed by less than 25% of the population, living in developed countries (18). In contrast, the dietary intake of animal protein is meager in developing countries, ranging from just 7 to 17 g/person/day, and contributing less than 2% of total energy (45, 46).

Even where the nutritional contribution is small, the production of livestock may have a significant effect on the economy (26, 47–49). For instance, livestock contribute to the national food supply by converting inedible or unpalatable plant material into milk, meat, and eggs. Sales of their products provide direct income to farmers, and create jobs for people on the land, in marketplaces, processing plants and other stages of the value chain (49). These same animals compete with people for food, especially grain fed to pigs and poultry (45). Livestock supply the world's population with less than 15% of its total dietary energy needs, but consume half the world's production of grain to do so (45, 47).

A total of 17 billion livestock are reared in three main types of production (47). Data from 2001 to 2003 suggest that: intensive systems provide 45% of the world's meat; grazing systems provide 9% of meat and 12% of milk; mixed crop–livestock systems supply 46% of meat, 88% of milk, and 50% of cereals (49). The latter offers efficiencies to sustainably increase production, but farmers may not be able to keep ahead of population growth, environmental change and the increasing demand for animal protein (26).

**Figure 1:**
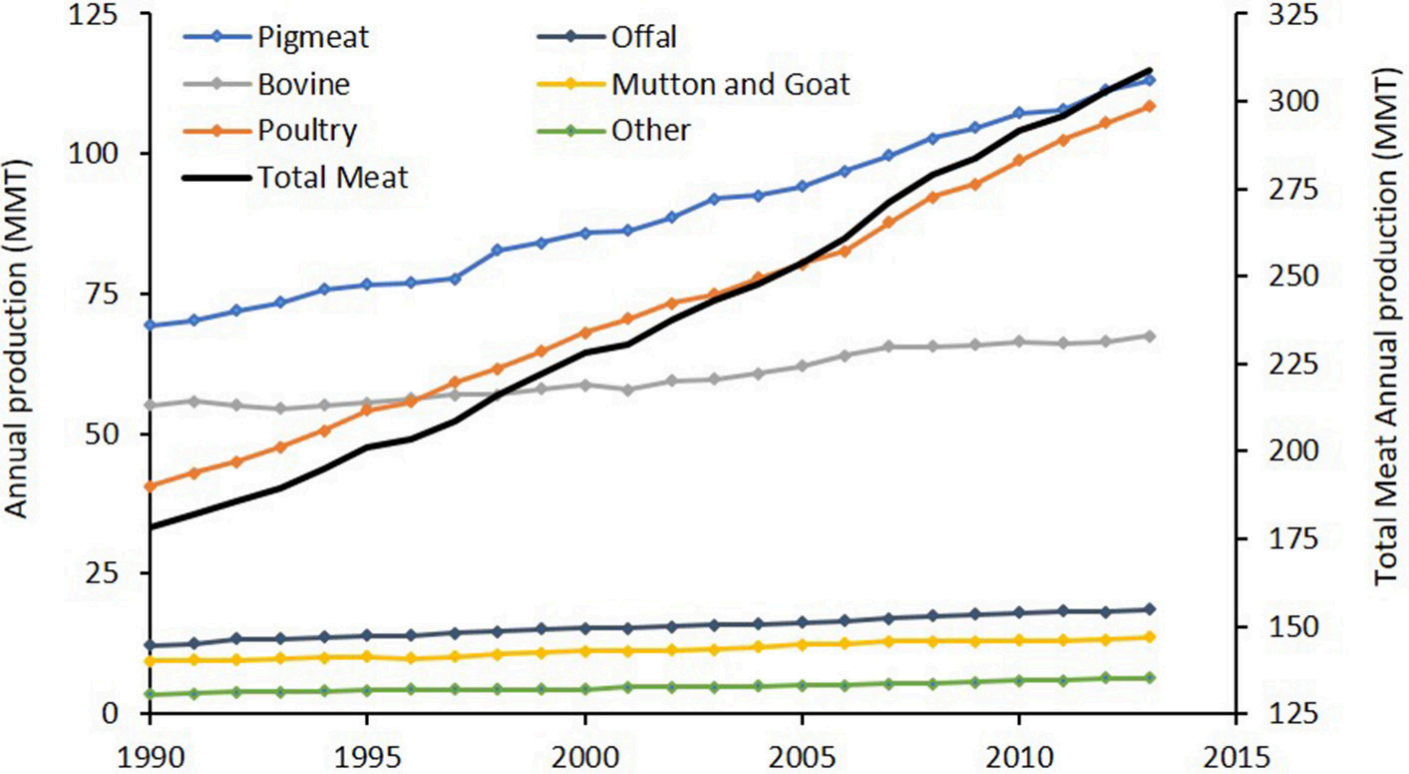
Global production statistics for various meats and offal in million metric tons (MMT), from 1990 to 2013 (18). Total meat does not include seafood or shellfish.

There has been a significant increase in aggregate meat production from terrestrial livestock over the past 20 years, from 178 MMT in 1990 to 330 MMT in 2016 (18). While population growth is partly responsible for the increased demand, there has been approximately 30% increase in per capita annual consumption (from 34 to 44 kg) which is likely the result of a nutrient transition accompanying rising incomes in developing countries (50). The profile of meats produced also changed markedly over time (Figure 1). Statistics from 1990 to 2013 show a 74% increase in total meat production, with poultry undergoing the largest individual change, increasing by 167% to contribute 35% of total meat production (18).

### What is consumed

There are two major challenges in estimating global meat consumption: insufficient data at community and household levels for people living in developing countries, and difficulty in accurately estimating meat composition in composite meals from food survey data collected in developed countries (51, 52). Food balance sheets (FBS) produced by the Food and Agricultural Organization of the United Nations (FAO) estimate at best the “average apparent consumption.” This leads to overestimates, as large volumes of material are lost during the processing of primary food products for retail sale and during household preparation (e.g., disposal of bones, cartilage, and fat) (25, 51). A more detailed portrayal of eating behavior can be gleaned from food surveys. The United Kingdom collects data on food purchases via the Living Costs and Food Survey (LCFS, formerly the Expenditure and Food Survey) (53). Its advantage over FBS is that it directly queries the household level, although it too may over-estimate actual consumption relative to purchasing (51). As an example, in 2013 the average person from the UK purchased ≈170 g of meat and offal each day (comprising 26 g red meat) (54). The FBS estimate for the UK was 220 g of meat available per person per day.

Meat availability varies around the world. The more developed countries average 130 g/person/day of which red meat (including pork, beef, veal, lamb, and goat) contributes 80 g (Table 1). By contrast, the least developed countries are consuming 25 g/person/day of red meat total, which is approximately one third of the global mean, and just an eighth of the average Australian (18).

**Table 1:** Average annual production of total meat and red meat only (bovine, pork, mutton, and goat), and its estimated contribution per capita during 2013 (18).

	**Production (MMT/year)**		**Availability (g/person/day)**	
**Location**	**Total**	**Red meat**	**Total**	**Red meat**
World	302	190	118	75
Least developed countries	11	7	39	25
**TOP CONSUMING COUNTRIES**				
Australia	3	2	318	186
Argentina	4	3	294	185
United States	37	21	315	176
New Zealand	0.5	0.3	278	174
Uruguay	0.3	0.2	225	148
Canada	3	2	249	148
Brazil	20	11	267	144

A broadly endorsed dietary recommendation for the general adult population is to not exceed 455–500 g of cooked lean red meat (and processed meat) per week, or approximately 87–107 g of raw meat per day (55–59). Based on this consensus, many developed countries have access to more meat than they need. There have been calls for a global rebalancing, imploring that those who eat too much animal-source foods should eat less and those who eat too little, should eat more (47). A target of 90 g of meat per person per day has been suggested, with half or less coming from red meat sources (60). Such goals are only part of the story however. Comparison of per capita consumption is a proxy for understanding each nation's ability to utilize meat to achieve acceptable nutritional standards (see below).

### What is wasted

**Figure 2:**
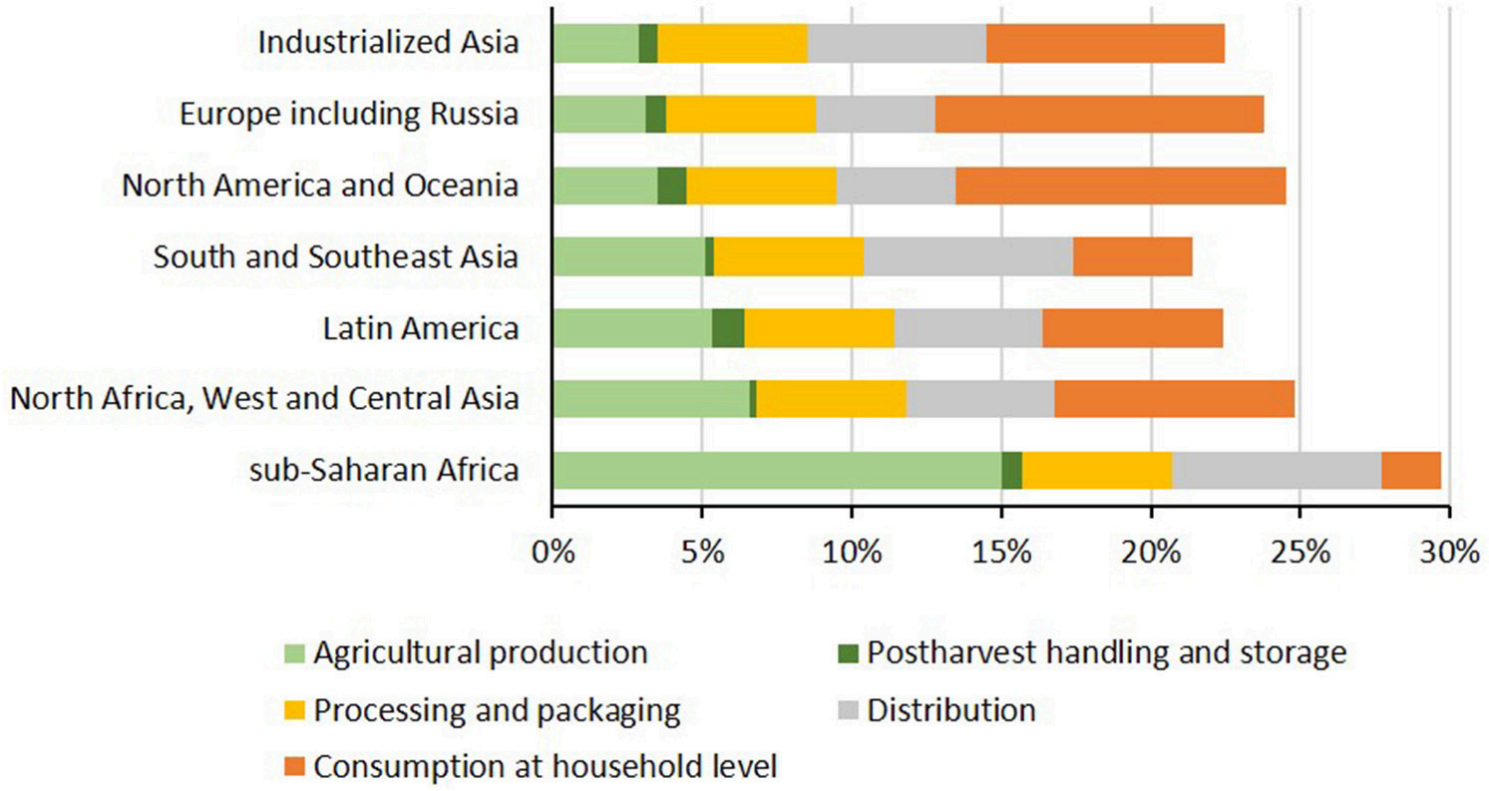
Percentage of meat lost and wasted from various stages of the food supply chain for different regions (62).

Losses and waste are generated throughout the meat supply chain, with significant differences in how these occur around the world (Figure 2). Less developed regions typically incur losses equally through the stages of the supply chain, with inadequate food-chain infrastructure and lack of investment in on-farm storage technologies being important vulnerabilities (24, 61). Sub-Saharan Africa loses an exceptional 15% of their meat supply during the initial agricultural production stage, due to high animal mortality from diseases and parasites. Losses in industrialized regions are modest during agricultural production, post-harvest handling, and storage as a consequence of good control of animal health during rearing and transportation to slaughter. Losses are more severe at the end of the chain, with large proportions of waste being generated by retailers and consumers (62). For example the Waste and Resources Action Programme estimated that 5% of lamb, 8% of beef, 12% of pork, and 21% of poultry purchased by consumers in the UK was discarded as “avoidable” waste, totaling 163,000 tons in 2012 (63).

Globally, 11% of meat is estimated to be lost during production, post-harvest handling, and storage and during processing and packaging, plus a further 12% during distribution and at the household consumer level. Due to the surplus of food produced in developed countries, the quantity wasted per capita is 5 to 10-fold greater than lower income regions. In Europe and North America that amounts to 18–30 kg of meat per person per year compared with sub-Saharan Africa and South/Southeast Asia at 2–5 kg based on data from 2013 (18, 62).

## Dietary choices and requirements

People select their food, including meat, to fulfill a variety of purposes beyond the merely functional (30). Freedom to make dietary choices is sometimes restricted, but factors that influence the quantity and type of food consumed can usually be categorized as (30, 64, 65):

1. Product-related: physicochemical properties, nutrient contents, sensory attributes, and functionality (convenience, availability, packaging, durability).
2. Consumer-related: demographic factors, metabolism (hunger, thirst), psychological dynamics (motives, attitudes, personality).
3. Environmental: economic, social (social group, family patterns, habits), cultural (traditions, religions), and context (place, time, and company associated with eating).

These factors are equally important in informing the dietary choices of flexitarians as well as other dietary demographic groups. However, for flexitarians, the environmental and social considerations outweigh others in their choices to reduce the quantity of meat they consume. For all consumers, food choices and dietary patterns in place today have developed in the context of the industrial revolution and subsequent introduction of a global food economy. Nutritional requirements of consumers however, are unlikely to have changed substantially since the Paleolithic era. The current orthodox standards for diet quality are the dietary reference intakes (DRI), which are a set of reference ranges based on observed relationships between nutrient intakes and indicators of adequacy and chronic disease in healthy populations. They are issued by the Food and Nutrition Board of the Institute of Medicine, National Academy of Sciences (66). Similar reference values are supported by national and international expert groups such as the Scientific Advisory Committee on Nutrition (SACN), and the European Food Safety Authority (67, 68). These provide the basis for nutritional guidelines set out by individual countries. Age and gender-specific recommendations have been published for intakes of macro and micro nutrients required to meet the needs of half the population (estimated average requirement, EAR), and/or the needs of 97% of the population (recommended dietary allowance, RDA). The values reflect what is considered an “adequate” diet, but defining an “optimal” diet remains a challenge (69).

### Adequate nutrition

Humans are omnivorous, opportunistic eaters. Incorporating meat and animal products is not a necessity for survival, but it facilitates achieving a complete and balanced diet, particularly in cultures and climates where food diversity is limited. General dietary advice tends to recommend eating plenty of vegetables and fruits, good fats, wholegrain carbohydrates and healthy sources of proteins (limiting consumption of red meat and excluding processed meat) (70). The many benefits of a plant-rich diet include its low energy density, high content of fiber, polyphenols, antioxidants and water, and usually low concentration of saturated fatty acids. However, wholly vegan and vegetarian diets are limited by physico-chemical impediments to digestibility, the presence of only inorganic (non-heme) iron that may be abundant but has low bioavailability, phytate chelation of essential elements such as zinc, deficiency in vitamin B12 that is derived almost exclusively from ruminant microflora, low content of omega-3 fatty acids (longer than the 18-carbons of alpha linolenic acid), and a risk of imbalance across indispensable (essential) amino acids (8).

The nutritional deficiencies and complications of a plant-based diet can be mitigated with animal products. Meat, offal, and marrow are nutrient-dense, and contain high quality digestible protein comprising a balanced profile of amino acids, essential micronutrients including iron (as heme and inorganic forms), selenium, zinc, and vitamins A, D, and B12 (8, 25, 71). Specific composition of meat and organs varies depending on animal species, age, sex, breed, and feed, and the butchery cut (72).

Most animal-derived proteins have high bioavailability when consumed. This is a key contribution to flexitarian diets. Advantages over equivalent quantities of plant proteins have been quantified experimentally, often using the Protein Digestibility-Corrected Amino Acid Score (PDCAAS) as a way to measure and rank ability to meet dietary requirements (73, 74). The technique tends to overestimate protein quality and some plant-based foods may appear to be more complete than they are (75). Recent advice is to replace PDCAAS with the Digestible Indispensable Amino Acid Score (DIAAS), which is based on ileal digestibility to better reflect the amounts of amino acids absorbed (76). Both systems have the weakness of focusing on discrete sources of protein and generating scores for individual foods. These can be unhelpful for designing and evaluating real-world diets where various protein sources are combined.

**Figure 3:**
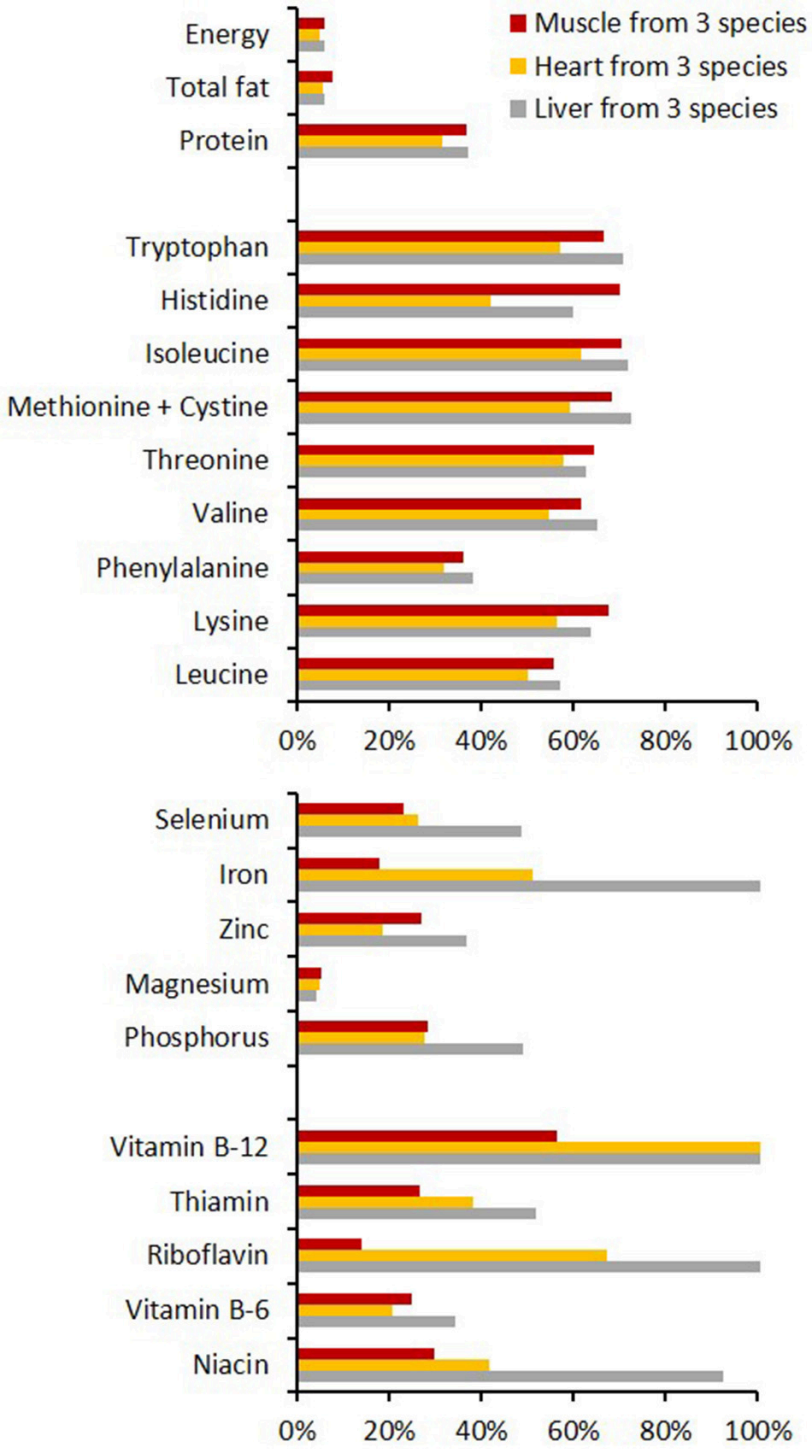
Typical content of key nutrients in lean muscle meat, heart and liver, shown here as the averaged values of lamb, beef, and pork sources (78) and expressed as a proportion of the adult male RDA (66) that would be provided by a 100 g raw serving. Items within nutrient classes are ordered by their increasing dietary requirement: amino acids 5–42 mg/kg bodyweight/day, minerals 55 μg−1,200 mg/day, vitamins 2.4 μg−14 mg/day. Calculations were based on the materials' raw native state, therefore water content is higher and nutrient concentrations are concomitantly lower than would be expected in the cooked versions of these foods.

**Figure 4:**
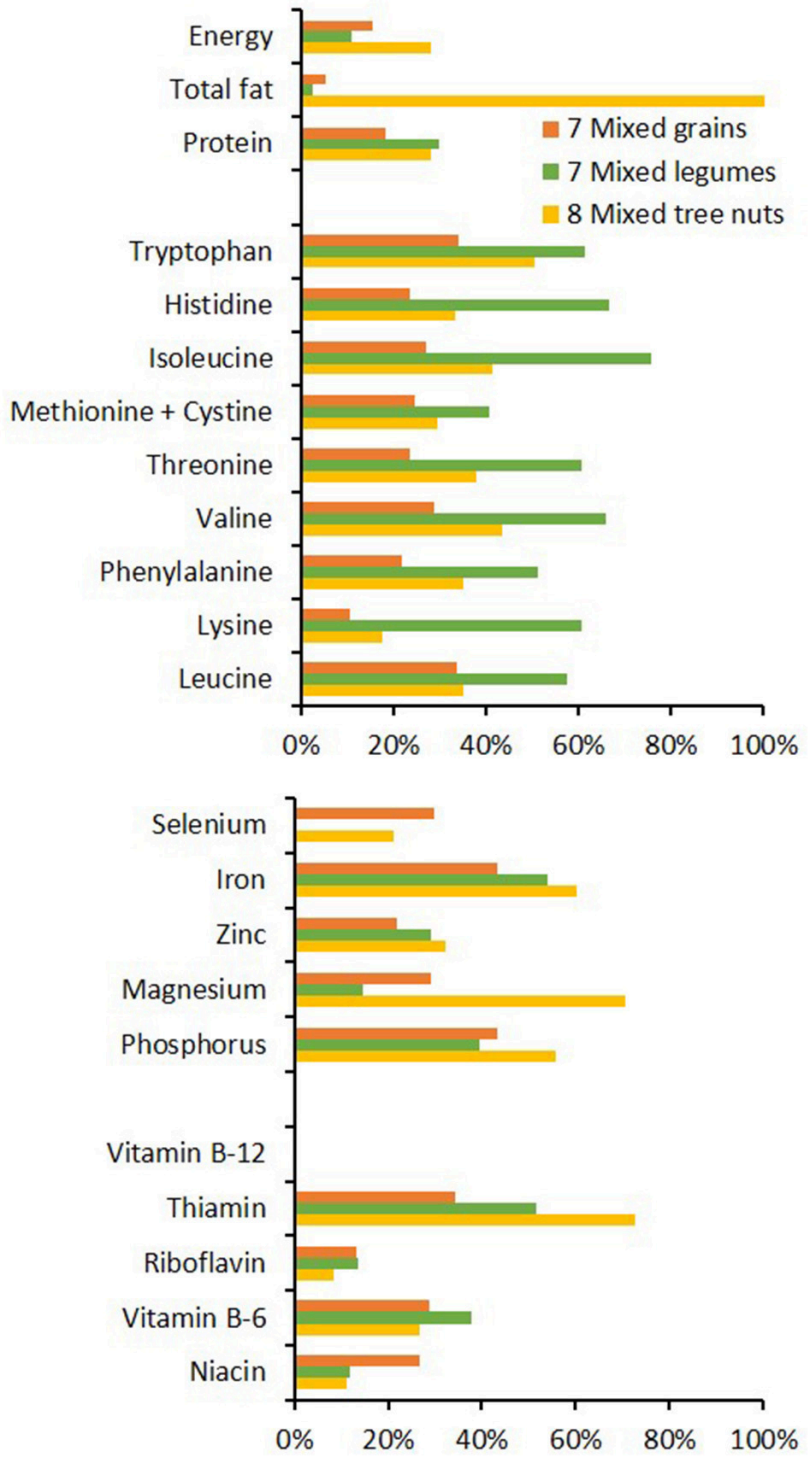
Typical content of key nutrients in grains, legumes and tree nuts, shown here as the averaged values of 7–8 sources (78) and expressed as a proportion of the adult male RDA (66) that would be provided by a 100 g raw serving. Items within nutrient classes are ordered by their increasing dietary requirement: amino acids 5–42 mg/kg bodyweight/day, minerals 55 μg−1,200 mg/day, vitamins 2.4 μg−14 mg/day. Calculations were based on the materials' raw native state, therefore water content is much lower and nutrient concentrations are concomitantly higher than would be expected in the cooked versions of these foods.

The chemical composition of meat and offal (as well as poultry, fish, eggs, isolated soy protein, and dairy foods) provides protein, fat and macro and micro nutrients in significant quantities relative to the proportion of energy (Figure 3) (77). Legumes, grains, nuts, seeds, and vegetables can be comparatively deficient in one or more of these amino acids and nutrients (Figure 4). The strategy for vegetarians is to combine cereals and legumes to get all of the indispensable amino acids. Direct comparison of animal and plant foods is confounded by the effects of cooking and exchange of water. Raw meat tends to lose water when cooked, which slightly increases the concentrations of its nutrients (“nutrient density”) per g consumed. By contrast most raw legumes and cereals gain water, which markedly decreases the concentrations of nutrients per g consumed.

**Figure 5:**
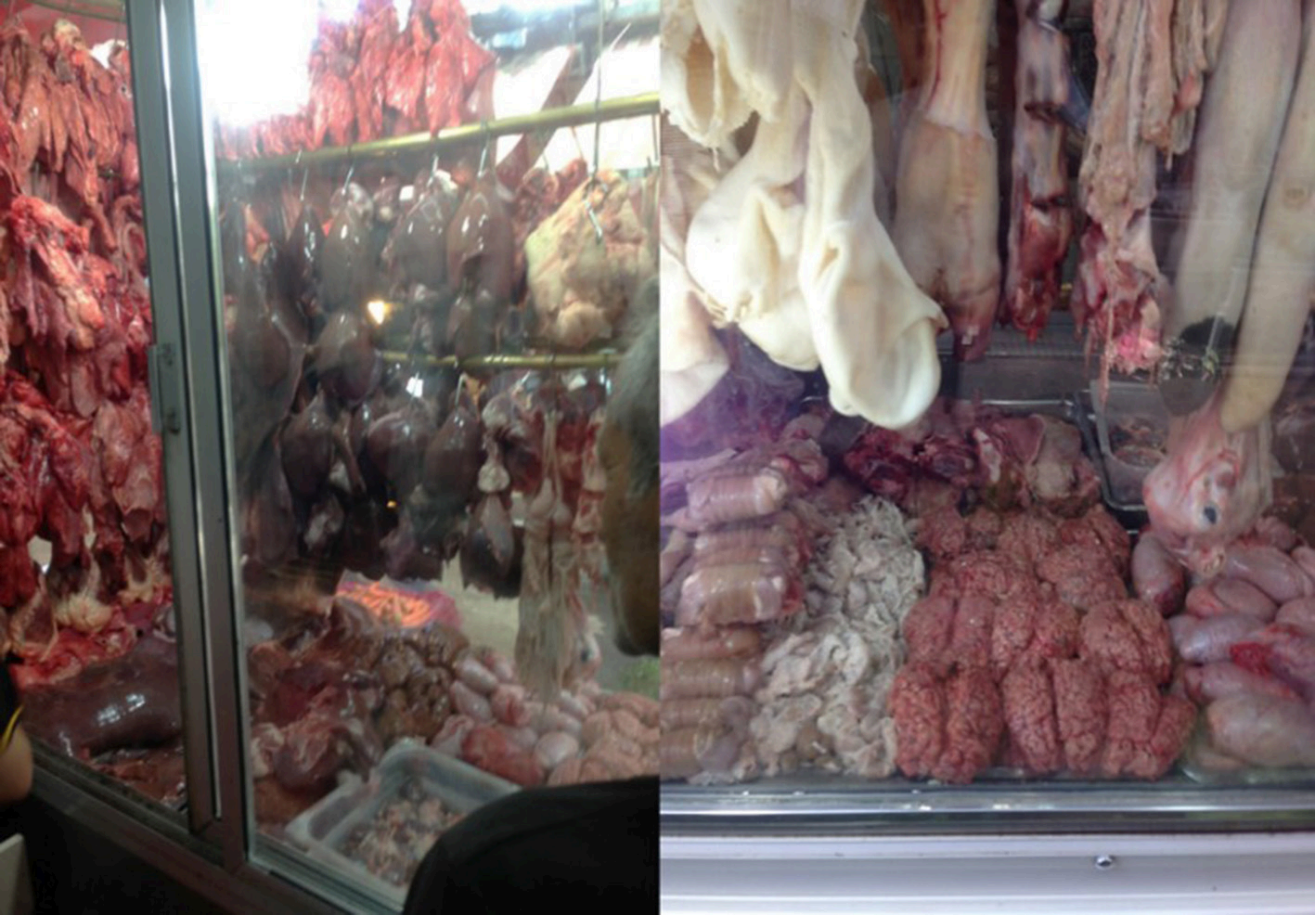
Organ meats on display in a retail cabinet in a market in Izmir Turkey. Almost all parts of the carcass are being utilized for human consumption. Small quantities of these nutrient-dense organ meats can significantly contribute to a flexitarian diet.

Most offal types tend to be richer in iron and vitamin B12 than lean muscle meat. They provide considerable quantities of indispensable amino acids as well as essential fatty acids (data not shown). In some parts of the world blood, liver, lung, heart, kidney, brain, spleen, and intestines are considered integral to the diet and can attract demand and prices greater than the muscle meat itself (Figure 5) (44, 79).

## Steps toward achieving food security

In order to feed the world while managing environmental impacts, meat production, and utilization must become more sustainable. This may be approached by diversifying the types of food we consume and obtaining the highest nutritional value from them through innovative product design.

### Greater diversity

There is increasing interest, research, and capitalization around using new or non-traditional animals for protein for human consumption (27). In addition to improving local biodiversity, broadening the range of available choices is a way to enhance food security. For instance in recent years there has been substantial intensification of production of buffaloes, camels, deer, goats, horses, and non-domesticated game meat (80). However, there are no limitations on the size or species of potential meat producers. Thoughtful, sustainable approaches to the development of wild and semi-domesticated animals of mammalian, avian, reptilian or amphibian origin should be considered (81).

Rather than continuing to farm introduced species of cattle and sheep, it may be prudent to shift the emphasis to species that have specifically adapted to local climates. Their resilience to harsh environmental conditions and performance with native, sometimes poor quality feed could be an advantage (80). For example camel meat consumed in the Middle East, North and Central Africa, and horse meat consumed in Europe, China, Korea, and Japan have similar nutritional profiles to beef or lamb (78, 82). Further, India produces and exports water buffalo meat, which tends to have lower fat content than beef, and a lower price (78, 83). These and other animals, including rabbits, deer, bison, and elk are used for meat production in various parts of the world and have similar nutritional profiles to lamb, beef, veal, and pork.

### Increased utilization

**Figure 6:**
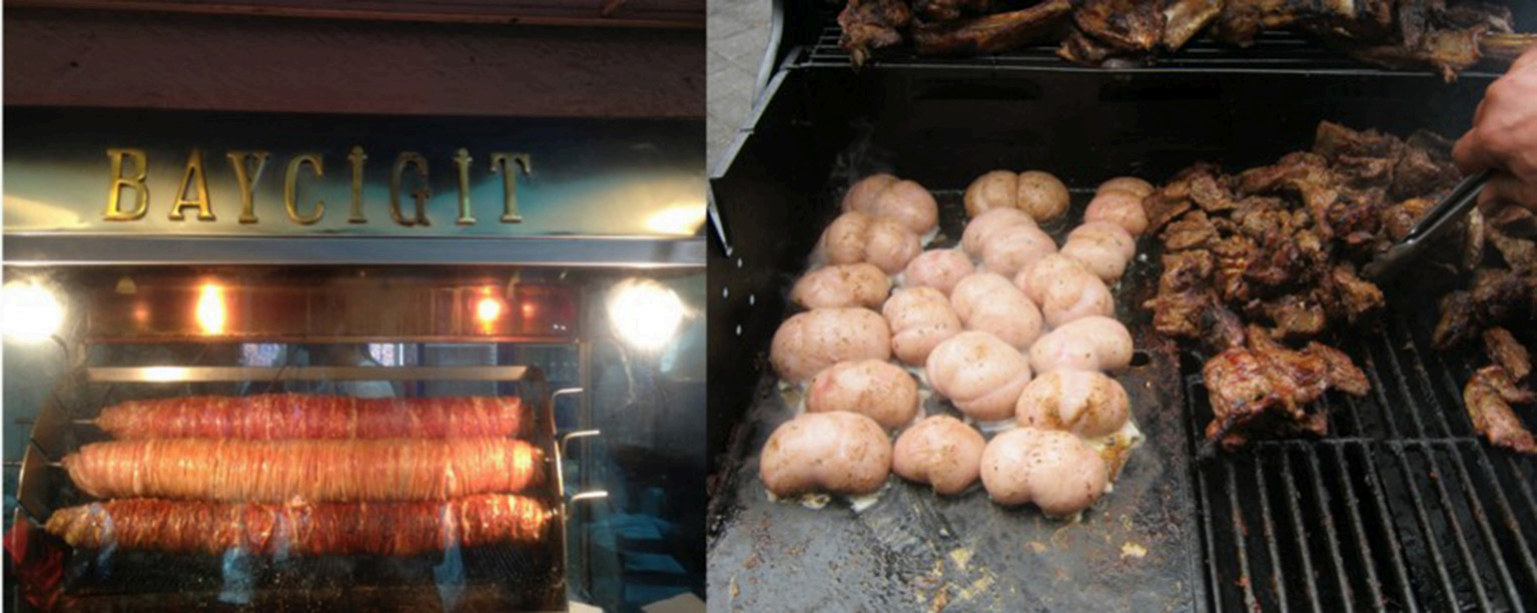
Forms of organ meat cooking and utilization. **(Left)** Shows sheep intestines being roasted for slicing and incorporating into fast-food meals in Izmir Turkey. **(Right)** Shows lamb meat and testicles barbecued side by side, demonstrating a possible combination of muscle and organ meats in a meal.

The first hurdle in sustainably producing more protein is to make maximum use of the animals already slaughtered. Offal is currently an underutilized resource. Its wider application into processed foods, ingredients and ready-to-eat and designer meals (particularly in markets where traditional offal cuisine is less appreciated) is a pathway toward sustainable meat consumption (Figure 6). In the USA and Canada, the cattle head meat, weasand, tongue root trim, and heart can be used in food formulations and clean-labeled as beef (84). Virtually all offal inspected in the USA is collected for sale, but up to 60% of it is exported. In the USA market, most offal is incorporated into minced beef, hot dogs and sausages, with small intestines from lamb and pork finding use as casings. Other edible offal such as tongues, livers, and pork ears are almost all exported, with chicken feet, pork tails, and beef tendons being processed and packaged like jerky in some Asian markets (84).

Red meat and offal are complex materials that can be broken down or deconstructed into their constituents and used as functional ingredients to create food with customized rheological, textural, and nutritional properties (85, 86). Lower value cuts already find use in sausages and pet food, but there has been considerable research into novel premium applications. For example proteins recovered from offal and blood may become functional ingredients in food (79, 87). Bioactive peptides that have antioxidant, antimicrobial, antihypertensive, or mineral binding properties have been reported (88). In addition, compounds in demand as nutritional supplements such as creatine, carnosine, carnitine, anserine, and taurine are highly enriched in animal-derived products. Other compounds of interest may be recoverable including conjugated linoleic acid, chondroitin sulfate, Coenzyme Q10, spermine, choline, lipoic acid, and glutathione (87).

New technologies have expanded the opportunities for repurposing meat and co-products. In the past few years, projects sponsored by Meat and Livestock Australia have attempted powderizing offal to produce shelf-stable capsules for combating malnutrition, and using meat powders for 3D food printing applications (89, 90). In this respect, meat along with other ingredients such as fruit, vegetables, legumes, cereals, and dairy can be combined in such a way as to enhance the aesthetic appeal of food, and improve digestibility and subsequent benefits of the food consumed—in a sense making the product greater than the sum of its parts (86).

Presenting meat in unexpected formats under new product categories will undoubtedly encounter technical, commercial, and cultural barriers, but the opportunities are substantial (86). Several novel food preparations have been investigated including bread, spaghetti, ice cream, yogurt and chocolate enriched with red meat protein (85, 91). Although these early studies were aimed at the elderly, the majority of these products have high levels of acceptability for people of all ages (85).

### Improved processing and distribution

Efficient production, processing, and distribution of food is paramount to achieving food security. There is no one-size-fits-all solution, and the successful, industrialized provisioning of meat is especially complex.

For developing countries this may require significant improvements to farming methods and market-led acquisition of new technological skills and knowledge (92). Increased mechanization, use of fertilizers and improved seeds may also raise productivity and reduce food loss. However, establishing food processing and storage infrastructure around the local mixed crop-livestock farming systems will be crucial if the maximum benefit from yields is to be realized. Such systems may not look like those of conventional Westernized countries. They may need to be smaller and more mobile and flexible to cater to both sparsely populated areas and overcrowded cities. In the case of abattoirs for instance, this could mean moving around to service neighboring regions (93), cope with low water inputs and labor, as well as being able to adequately handle a variety of different sized animals of different species. In parallel, new ways of preserving and storing food must be introduced. Each storage system must be considered in light of its environment, taking the best of science and local wisdom to function optimally within the culture, climate, and resources available.

Introduction of better infrastructure and processes will enhance food security only when there is a stable political environment in which such markets can thrive. Creating a more efficient global food supply chain is also going to require policymakers to consider ways to establish open markets or lower agricultural import tariffs so that food waste from developed regions can be directed toward countries that have insufficient food (94).

### Education to reduce waste

There will be little progress in reducing food waste without first a change in mindset—how we view, understand and acknowledge food (24). Some food losses stem from cavalier attitudes, but others are simply misguided good intentions. A recent survey conducted by Kantar Public found that a substantial number of people have misconceptions about how to safely freeze food, and are instead disposing of it unnecessarily (95). Two thirds of respondents had thrown away food in the month prior. At least 30% had discarded food simply because they had purchased too much, and 40% believed food could only be frozen on the day it was purchased in order to be safe, with a similar proportion believing it dangerous to freeze cooked meat. In addition, 36% of the people surveyed believe that food could become unsafe to eat while in the freezer.

Education can raise awareness of the social and environmental impact of waste (92). After the results of the Kantar Public survey were published, the UK Food Standards Agency responded by using Food Safety Week to focus on how to use freezers to reduce food waste (96). By continuing to spread positive messages about the benefits of dietary flexibility, animal health and personal health, more people may shift their diet to a more environmentally sustainable one (97, 98).

## Meat industry responses to flexitarianism: motivations and actions

There are many reasons people adopt a flexitarian diet. For much of the world's population, and especially in developing countries, flexitarianism is compelled by circumstance and is the default lifestyle. In affluent societies, people can respond as they wish to messages about health, nutrition, weight control, environmental sustainability, food security, animal welfare, and other ethical concerns (99–101). The meat industry regards those issues as relevant too, which is why the industry should provide leadership in facilitating consumers' dietary choices while maintaining its primary responsibility of keeping meat safe, available and firmly on the menu.

### Broaden the meat consumer base

The meat industry should redefine its consumer base by regarding everyone who is not an obligate vegan as a flexitarian, and work toward understanding the interests and requirements of this broad target group. A recent Dutch survey found that 77% of consumers considered themselves to be meat-reducers and not avoiders (100). It would be efficient and wise for the industry to build a strategy around that demographic, to ensure their needs are met and to keep them consuming meat, rather than risk losing them to veganism. The strategy must acknowledge the diverse expressions of flexitarianism. For example cluster analyses segmented all the meat eaters into conscious, unconscious and extravert flexitarians, disengaged meat-eaters, and meat-lovers (100, 102). These categories could be catered for profitably and sustainably if the meat industry acts proactively.

### Re-brand the meat industry

To better serve flexitarianism and sustainability and to leverage the inescapable trend of meat analogs and substitutes, the traditional meat industry should re-brand itself into a Meat and Complementary Products Industry. This would enable bolder promotion of co-products and so encourage their production and utilization. And it would “allow” the industry to get into the business of producing meat-alternatives and directing the narrative, rather than leaving that to competitors. This updated Industry could also promote greater transparency about the amounts and sources of protein in products and meals.

### Price meat right

Most of the meat produced by industrialized agriculture in developed countries is too cheap. Competition within the industry has pushed margins down and farmers are forced to pursue volume rather than quality. The number of animals required just to keep up revenue has turned a precious resource into a commodity that many consumers take for granted. Pricing meat accurately for its true social and environmental cost would reduce the in-market quantities. This is actually a better fit for flexitarianism, closing the gap between those who are forced to live the lifestyle and those who do it by choice. It would also maintain farmers' income while rearing fewer animals and motivate more care regarding losses and waste. Resetting prices might come about through government intervention, if conditions were imposed on the social license to operate. Or farmers and producers in a market might create a cartel akin to industries such as oil and gemstones, and take more control of supply and demand.

### Supply flexitarian foods and meal solutions

The meat industry should increase its involvement with supplying food and meal solutions rather than meat products alone. Meals in formats of ready-to-cook, ready-to-heat and ready-to-eat usually include meat, meat alternatives or other animal proteins with vegetables and starches. Flexitarian consumers will already be familiar with the concepts of ingredient complementarity and advantageous combining, and would be comfortable with small proportions of meat. These prepared meals could also be a new vehicle for utilization of organ meats, which the industry could promote nutritionally and gastronomically.

## Conclusions

The accessibility of meat in the Westernized world is a privilege taken for granted by many. At this stage, meat industries rely primarily on high production efficiency, placing significant strain on our natural resources. Adopting a flexitarian diet has become a socially innovative means for individuals to reduce their impact on the environment, and collectively support the de-intensification of the livestock industry.

When consumed in moderation, meat and offal has an important role in maintaining good health through its supply of nutrients, including nutritionally complete proteins, bioavailable iron, zinc, and selenium and the exclusive source of vitamin B12. The adoption of a flexitarian diet adds the benefits more high fiber, plant-based foods, with the nutritional durability of an omnivorous diet.

In order to adequately meet everyone's dietary needs it is important that meat is more equitably distributed amongst the world's population and its sale as a commodity product is curtailed. Extensive on-going research into post-harvest storage technologies and packaging for improving the shelf life of perishable foods will be essential. This is particularly true for developing countries that experience the greatest food losses due to inadequate resources.

Total meat production must be reduced in the industrialized world, with a higher value obtained through a focus on increasing the diversity of species consumed, and incorporating a greater proportion of edible offal into ready-to-eat meals and designer foods. To accommodate flexitarianism, the red meat value chains will need to proactively engage and adapt to the changing consumer base, with the entire food sector involved in developing innovative processing, product matching, packaging, presentation, and distribution solutions. It is anticipated that the red meat industry will respond enthusiastically to the rising trend of flexitarianism, and that it will view this as an opportunity to produce higher value and niche products, rather than commodity meat.

## Author contributions

TH prepared the initial manuscript, figures, and tables. This was subsequently edited by SK in consultation with MF. Final edits were made collectively by all three authors.

### Conflict of interest statement

The authors declare that the research was conducted in the absence of any commercial or financial relationships that could be construed as a potential conflict of interest.
